# Patient complaints in radiology: 9-year experience at a European tertiary care center

**DOI:** 10.1007/s00330-019-06158-z

**Published:** 2019-03-22

**Authors:** Pieter F. van den Berg, Derya Yakar, Andor W. J. M. Glaudemans, Rudi A. J. O. Dierckx, Thomas C. Kwee

**Affiliations:** Medical Imaging Center, Department of Radiology, Nuclear Medicine and Molecular Imaging, University Medical Center Groningen, University of Groningen, Hanzeplein 1, P.O. Box 30.001, 9700 RB Groningen, The Netherlands

**Keywords:** Hospital-patient relations, Patient-centered care, Patient satisfaction, Radiology

## Abstract

**Objective:**

To determine the frequency, nature (using standardized coding taxonomy), and temporal trends of patient complaints about the radiological service provided in a European tertiary care center.

**Methods:**

This retrospective study included all written patient complaints received by the department of radiology of a European tertiary care center within a 9-year period.

**Results:**

A total of 94 written patient complaints were included. Overall complaint frequency was 14.4 per 100,000 radiological procedures. Complaint frequencies per 100,000 procedures were 103.7 for interventional radiology, 13.9 for MRI, 6.9 for ultrasonography, 6.5 for CT, 4.5 for fluoroscopy, and 1.2 for conventional radiography. Interventional radiology received significantly more complaints than all other radiological procedures (*p* < 0.001), and cross-sectional imaging (CT, MRI, and ultrasonography) received significantly more complaints than conventional radiography (*p* < 0.001). Fifty-three (56.4%) complaints belonged to the clinical domain, 22 (23.4%) to the relationships domain, and 19 (20.2%) to the management domain. Quality (34.0%), safety (22.3%), timing and access (18.1%), and communication (18.1%) constituted almost all complaint categories. Patient journey (19.1%), delays (18.1%), communication breakdown (16.0%), errors in diagnosis (11.7%), quality of care (9.6%), treatment (6.4%), and staff attitudes (2.1%) constituted almost all complaint subcategories. Annual frequency of complaints decreased over time (Mann-Kendall tau = − 0.429), although not significantly (*p* = 0.174).

**Conclusion:**

Written patient complaints directed to a department of radiology at a European tertiary care center are relatively few in number and have not shown a temporal increase. Knowledge of sources of patient dissatisfaction may help to reduce the number of patient complaints and improve patient care.

**Key Points:**

• *Approximately 14.4 written patient complaints per 100,000 radiological procedures are filed in a European tertiary care center, and they have not increased over a 9-year period.*

• *Written patient complaints most frequently involve interventional radiology, and the main complaint categories are quality (34.0%), safety (22.3%), timing and access (18.1%), and communication (18.1%).*

• *Knowledge of the nature of and circumstances under which patient complaints arise may reduce their number and improve patient care.*

## Introduction

Patient satisfaction is a crucial indicator that reflects the quality of healthcare [1]. The importance of assessing patient (dis)satisfaction has been acknowledged as a core component of practicing patient-centered radiology, a concept in which radiology healthcare providers partner with patients and families to identify and satisfy patients’ needs and preferences [2].

Remarkably, there is limited literature on the frequency and causes of patients’ complaints about the services of a radiology department. A study by Salazar et al [3] reported an overall incidence of unsolicited written complaints per radiologic procedure of 2.38 per 100,000, and that most of these complaints (60.1%) were due to failure to provide patient-centered care. Other studies on this topic in the field of radiology are lacking. Furthermore, the study by Salazar et al [3] was performed in a tertiary care center in the USA, and their results may not be applicable to a European setting due to differences in healthcare provision indicators and socio-economic patient variables [4]. In addition, they did not use a standardized coding taxonomy to analyze patient complaints [3], making comparisons with other studies difficult. Moreover, they reviewed complaints that were received between 1999 and 2010 [3]. Essential issues that have changed since then are the increasing number of (cross-sectional) radiologic examinations in the Western world over the years [5], and patients may generally have become more demanding due to phenomena such as cultural shifts towards patients as consumers and the internet-informed patient [6]. This underlines the need for updated data.

The purpose of this study was to determine the frequency, nature (using previously developed standardized coding taxonomy [7]), and temporal trends of patient complaints about the radiological service provided in a European tertiary care center.

## Materials and methods

### Study design

This retrospective study was approved by the local ethics committee (IRB number: 201800207) and the requirement for informed consent was waived. The University Medical Center Groningen is a tertiary care center that provides primary and specialty care to over two million people in the northeast of the Netherlands. The department of radiology has a digital archive in which all written patient complaints (both e-mails and letters, whether from the patients themselves, their relatives, or other representatives) are stored that were received within a consecutive 9-year period (January 2010 to December 2018). According to the institutional protocol, all written patient complaints are received by or should be forwarded to the independent complaints officer. Each complaint is then sent to the head(s) of the department(s) involved, who in turn respond(s) with a letter directed to the patient via the complaints officer. Complaints received by another department which include issues relevant to radiology are passed on to the head of the department of radiology. All patient complaint letters were included for analysis in this study, except if the complaint was not related to a procedure that was performed at the department of radiology, or if it was unclear if this was the case. Financial compensation was given for travel costs, hotel stay (for patients who had to travel from far away for a radiological procedure that was scheduled early in the morning), and missed income on the day of the radiological procedure, upon specific request by the patient and if considered justified by the head of the department of radiology.

### Data extraction

All patient complaints were unsolicited and in non-standardized formats. A research fellow (P.F.v.d.B.) reviewed all patient complaints to retrieve the year in which the complaint was filed, age and gender of the patient, patient’s hospital status (inpatient, outpatient, or emergency department), type of radiological procedure (conventional radiography, fluoroscopy (performed by non-interventional radiologists outside the dedicated interventional radiology suite), ultrasonography, CT, MRI, or interventional radiology), specialty of the primary treating physician(s), if the complaint was solely directed to the department of radiology or if it was also directed to other departments (i.e., shared complaint), and whether or not a financial compensation was given by the hospital. All complaints were analyzed and interpreted using standardized coding taxonomy that was recently developed by Reader et al [7] (Table 1). This coding taxonomy for patient complaints uses three domains: “clinical” (complaints on the safety and quality of clinical care), “management” (complaints related to the management of the healthcare organization), and “relationships” (complaints about healthcare staff-patient relationships) [7]. The clinical domain is divided into the categories “quality” and “safety,” the management domain is divided into the categories “institutional issues” and “timing/access,” and the relationships domain is divided into the categories “communication,” “humaneness/caring,” and “patient rights” [7]. Categories are further divided into 26 subcategories, which are described in detail by Reader et al [7]. One complaint letter can contain multiple domains, categories, and subcategories. Subsequently, the severity of each complaint category was classified as “low,” “medium,” or “high,” according to Gillespie et al [8]. Note that severity ratings are independent of outcomes (i.e., harm) and not comparable across problem categories [8]. If one complaint letter contained different complaint categories with different severity ratings, the highest level of severity was recorded [8].

**Table 1:** Patient complaint taxonomy as adapted from Reader et al [7]

Domains	Categories	Subcategories	
Clinical	Quality	Examination	Inadequate patient examination by clinical staff
		Patient journey	Problems in the coordination of treatment in different services by clinical staff
		Quality of care	Substandard clinical/nursing care
		Treatment	Poor, or unsuccessful, clinical treatment
	Safety	Errors in diagnosis	Erroneous, missed, or slow clinical diagnosis
		Medication errors	Errors in prescribing or administering medication
		Safety incidents	Events or complications that threatened the safety of patients
		Skills and conduct	Deficiencies in the technical and non-technical skills of staff that compromise safety
Management	Institutional issues	Bureaucracy	Problems with administrative policies and procedures
		Environment	Poor accommodation, hygiene, or food
		Finance and billing	Healthcare-associated costs, or the billing process
		Service issues	Problems with hospital services for supporting patients
		Staffing and resources	Inadequate hospital staffing and resource levels
	Timing and access	Access and admission	Lack of access to services or staff
		Delays	Delays in admissions or access to treatment
		Discharge	Early, late, or unplanned discharge from the hospital
		Referrals	Problems in being referred to a healthcare service
Relationships	Communication	Communication breakdown	Inadequate, delayed, or absent communication with patients
		Incorrect information	Communication of wrong, inadequate, or conflicting information to patients
		Patient-staff dialogue	Not listening to patients, lack of shared decision-making, and conflict
	Humaneness/caring	Respect, dignity, and caring	Rude, disrespectful, or insensitive behaviors to patients
		Staff attitudes	Poor attitudes towards patients or their families
	Patient rights	Abuse	Physical, sexual, or emotional abuse of patients
		Confidentiality	Breaches of patient confidentiality
		Consent	Coercing or failing to obtain patient consent
		Discrimination	Discrimination against patients

### Statistical analysis

Characteristics of patients who filed the complaints; specialty of the primary treating physician(s); sharing of complaints with other departments; distribution of complaints among the different domains, categories, and subcategories according to Reader et al [7]; severity of complaints according to Gillespie et al [8]; and financial compensation were descriptively analyzed. Frequencies of complaints per type of radiological procedure were compared using a chi-square test with the Bonferroni correction. The presence of a temporal trend of complaint frequencies over the years was assessed using the Mann-Kendall test. Complaint frequencies were only analyzed for the years 2010 to 2017, because the hospital deployed a new electronic medical record software application in 2018, from which reliable production numbers could not yet be extracted. *P* values less than 0.05 were considered statistically significant. Statistical analyses were performed using R version 3.5.2 software (R Foundation for Statistical Computing).

## Results

### Patients and circumstances

A total of 96 complaint letters were submitted between January 2010 and December 2018.

Two complaint letters were excluded, because it was uncertain if they were related to a procedure that was performed at the department of radiology (both complaint letters were related to the placement of a central venous catheter without any record that this procedure was performed at the department of radiology). The 94 remaining complaint letters that were finally included concerned 44 male patients, 49 female patients, and 1 patient whose gender remained unclear, with a mean age of 48 ± 22.0 years (range, 6 months–79 years). Sixty-one (64.9%) of patients’ complaints were related to an outpatient setting, 22 (23.4%) to an inpatient setting, and 11 (11.7%) to an emergency department setting. Sixty-two (66%) of these complaints were filed by patients themselves, 17 (18.1%) were filed by family members, 8 (8.5%) were received through the Prevention Recovery Information System for Monitoring and Analysis (PRISMA)-Medical method [9], 4 (4.3%) were filed by other medical doctors, and 3 (3.2%) were filed by the partner of the patient. Treating specialty was most frequently surgery (19.1%), followed by orthopedics (12.8%) and emergency medicine (10.6%). Fifty-one (54.3%) of complaints were shared with other departments, and 43 (45.7%) were solely directed to the department of radiology. The department of surgery was most frequently involved in a shared complaint (33.3%), followed by the departments of orthopedics (17.6%) and pediatrics (15.7%).

### Frequency of complaints and comparisons among different radiological procedures

**Fig. 1:**
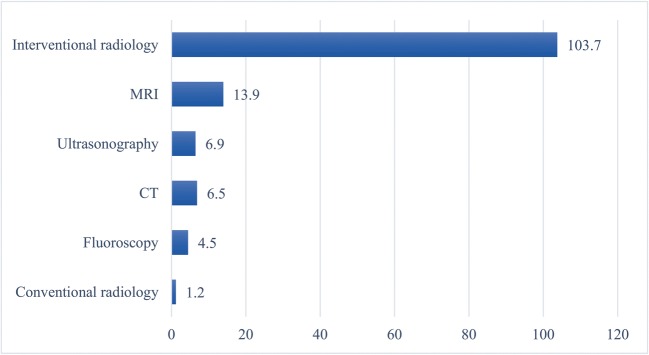
Number of complaints per 100,000 procedures performed (between 2010 and 2017), for interventional radiology, MRI, ultrasonography, CT, fluoroscopy, and conventional radiography separately

Eighty-nine complaint letters were filed between 2010 and 2017, in which a total of 618,482 radiological procedures were performed, corresponding to an overall complaint frequency of 14.4 per 100,000 radiological procedures. Frequencies of complaints per 100,000 procedures were, in order of decreasing frequency, 103.7 (95% confidence interval [CI], 74.6–144.2) for interventional radiology, 13.9 (95% CI, 9.0–21.5) for MRI, 6.9 (95% CI, 3.9–12.0) for ultrasonography, 6.5 (95% CI, 3.8–11.1) for CT, 4.5 (95% CI, 1.2–16.2) for fluoroscopy, and 1.2 (95% CI, 0.7–2.1) for conventional radiography (Fig. 1). Interventional radiology received significantly more complaints than all other radiological procedures (*p* < 0.001). All cross-sectional imaging modalities (CT, MRI, and ultrasonography) also received significantly more complaints than conventional radiography (*p* < 0.001). Other complaint frequencies did not differ significantly from each other (Table 2).

**Table 2:** Pairwise comparisons of frequency of complaints (per 100,000 procedures) among the different radiological procedures

	Fluoroscopy	Ultrasonography	CT	MRI	Interventional radiology
Conventional radiography	*p* = 0.965^a^	*p* < 0.001^a^	*p* < 0.001^a^	*p* < 0.001^a^	*p* < 0.001^a^
Fluoroscopy	–	*p* = 1.000^a^	*p* = 1.000^a^	*p* = 1.000^a^	*p* < 0.001^a^
Ultrasonography	–	–	*p* = 1.000^a^	*p* = 0.716^a^	*p* < 0.001^a^
CT	–	–	–	*p* = 0.407^a^	*p* < 0.001^a^
MRI	–	–	–	–	*p* < 0.001^a^

^a^Calculated with *z*-test for proportions and post hoc Bonferroni correction

### Sources and severity of patient complaints

**Fig. 2:**
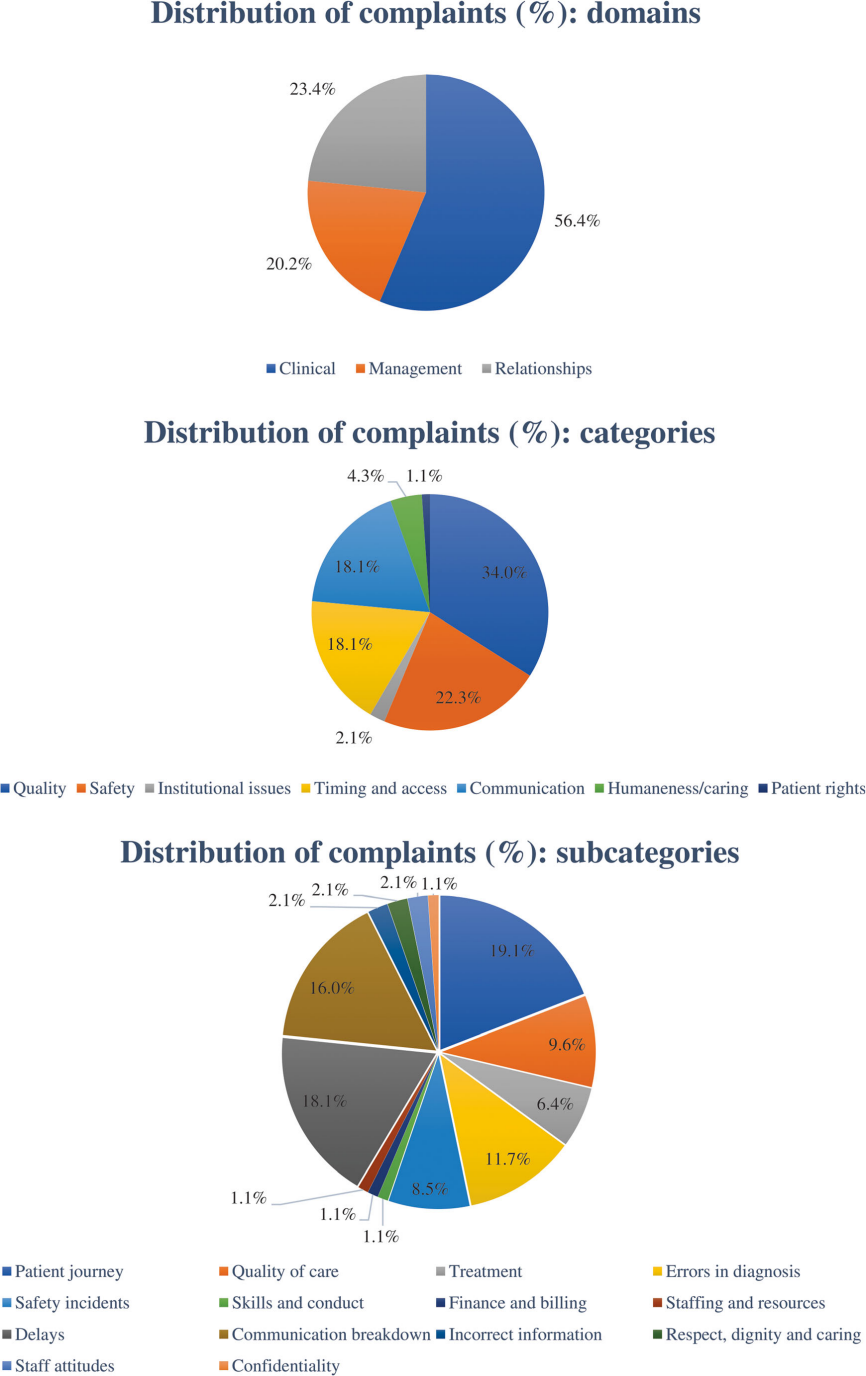
Distribution of complaints among the different domains, categories, and subcategories according to the patient complaint taxonomy by Reader et al [7]

Figure 2 shows the distribution of complaints among the different domains, categories, and subcategories according to the patient complaint taxonomy by Reader et al [7]. Fifty-three (56.4%) of complaints belonged to the clinical domain, 22 (23.4%) to the relationships domain, and 19 (20.2%) to the management domain. Quality (34.0%), safety (22.3%), timing and access (18.1%), and communication (18.1%) constituted almost all complaint categories. Patient journey (19.1%), delays (18.1%), communication breakdown (16.0%), errors in diagnosis (11.7%), quality of care (9.6%), treatment (6.4%), and staff attitudes (2.1%) accounted for almost all complaint subcategories. Complaint severity ratings were low, medium, and high, in 44 (46.8%), 28 (29.8%), and 22 (23.4%) of complaints, respectively.

### Temporal trends

**Fig. 3:**
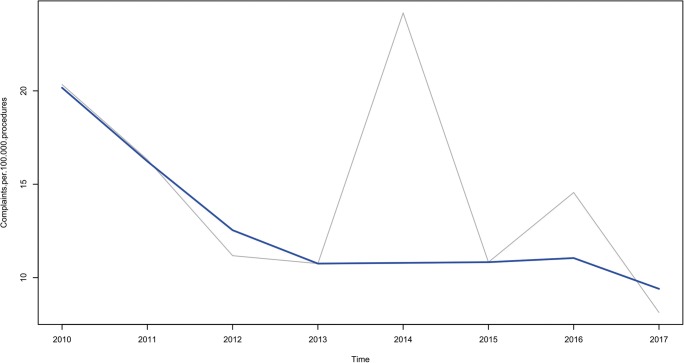
Number of complaints per 100,000 radiological procedures per year (gray line) with non-parametric LOESS fit in blue (Mann-Kendall tau of − 0.429; *p* = 0.174)

Figure 3 shows the annual frequencies of complaints that were filed. Between 2010 and 2017, the annual frequency of complaints decreased over time with a Mann-Kendall tau of − 0.429, but this did not reach statistical significance (*p* = 0.174).

### Financial compensations

Financial compensation was granted in 8 (8.5%) cases. Data regarding the amount of financial compensation was available in 5 of 8 cases. The mean financial compensation in these 5 cases was €94.69 ± 93.41 (range, €35.00–€253.46).

### Case examples

An example of a high-severity complaint concerned a 37-year-old woman who underwent an interventional radiology procedure (sclerotherapy) for the treatment of an arteriovenous malformation in the right lower arm. After treatment, she developed intense and increasing pain. Before, during, and after the treatment, the communication with the treating staff was experienced as poor.

An example of a low-severity complaint concerned a 47-year-old man who was supposed to undergo a CT scan of the abdomen because of autoimmune hepatitis, before his annual checkup at the department of gastroenterology. However, due to poor communication and planning, it was not possible to perform the CT scan before the checkup, as a result of which the value of the consultation with the gastroenterologist was experienced as considerably diminished.

## Discussion

The results of this study show that patients infrequently file a written complaint related to a radiological procedure that they have undergone at a tertiary care institution in Europe, with an overall complaint frequency of 14.4 per 100,000 radiological procedures. Moreover, the complaint frequency did not increase over time. In addition, nearly half of all complaints were of low severity. Although these results appear reassuring, it is imperative to keep the number of patient complaints to a minimum in light of the concept of patient-centered radiology and the growing trend towards more detailed public reporting of patient satisfaction data with benchmarking and links to financial reimbursements [2].

Interestingly, the majority of complaints were from outpatients and were shared complaints. The former cannot be explained, but the latter is plausible given the role of radiology as a supporting specialty. It is also interesting that the departments of surgery and orthopedics were most frequently involved, both as the primary treating specialty and as co-recipient of the complaint. This is in line with the results of a study by Tibble et al [10] that reported the rate of patient complaints to be 2.3 times higher for surgeons than for other (non-surgical) physicians. In addition, male surgeons were reportedly at a higher risk of complaints, as were specialists in orthopedics, plastic surgery, and neurosurgery [10]. Tibble et al [10] speculated that this elevated risk arises partly from involvement in surgical procedures and treatments, but also reflects wider concerns about interpersonal skills, professional ethics, and substance use.

Among all radiological procedures, interventional radiology was by far most susceptible to patient complaints. This is probably related to the more invasive nature of interventional radiology, with associated risk of complications and side effects. Another issue is that patient-physician communication may sometimes be compromised because of the higher time and work pressure for interventional radiologists who frequently deal with urgent and/or technically complicated procedures [3, 11]. In addition, it should be noted that in our institution, pre-intervention radiologist-patient consultations are only held for elective neuro-interventional procedures. Furthermore, the waiting time for some elective interventional radiology procedures (which could be up to several months) was also a common trigger for complaints in the present study.

Patients also filed significantly more complaints related to cross-sectional imaging (CT, MRI, and ultrasonography) than to conventional radiography. In general, planning, acquisition, and interpretation of cross-sectional imaging modalities are more complex and time-consuming than conventional radiography, and therefore more prone to adverse incidents and errors that may be perceived by the patient as below standard care. It can also be speculated that patients who undergo cross-sectional imaging generally have an a priori worse condition and more frequently have a more serious underlying disease. Furthermore, a study by Ollivier et al [12] showed that the vast majority of patients (73%) experienced their CT and MRI scans as distressing, both due to the scan procedure itself and due to fear of the results. These patient and scan-related factors may potentially lower the threshold for patients to complain.

According to patient complaint taxonomy [7], most complaints were related to the clinical domain, followed by the management and relationships domains. Quality, safety, timing and access, and communication comprised the far majority of complaint categories, and these targets should be prioritized with respect to both staff education and incorporation into continuous improvement systems (quality circles, total quality management, plan do act, Kaizen, etc.) [13]. Written patient complaints provide a valuable input for such continuous improvement systems. On the other hand, actively assessing patient (dis)satisfaction in the radiology department by means of routine patient surveys may perhaps be more desirable, because it provides a much broader and systematic view of how patient-centered radiology is delivered and may actually prevent patient complaints. However, except for the Press Ganey patient satisfaction survey for radiography and US performed in the outpatient setting [2], standardized and validated survey instruments for other imaging modalities and clinical settings are currently lacking.

Only one previous study, by Salazar et al [3], evaluated radiology-related patient complaints. This study was performed at Massachusetts General Hospital and comprised the period 1999–2010, in which 153 complaints were filed [3]. Their complaint frequency per 100,000 procedures was lower (2.38) than ours (14.4), but a common finding was the significantly higher incidence of complaints associated with interventional radiology procedures [3]. Most of their complaints (60.1%) could be grouped under the denominator “failure to provide patient-centered care,” but direct comparison with the present study is difficult because Salazar et al [3] did not use the standardized coding taxonomy developed by Reader et al [7]. Furthermore, their results may not be applicable to hospitals outside the USA due to differences in healthcare provision indicators and socio-economic patient variables [4], as mentioned before.

This study had some limitations. First, it was performed in a tertiary care university medical center in Europe, and the results may be different in non-European and non-academic hospitals with other patient populations. More personalized contacts between radiologists and patients may decrease complaint frequency [14], but this requires further investigation. Second, only unsolicited patient complaint letters were available for analysis. Many unhappy patients may not formalize their complaints, while they would express dissatisfaction in a survey or in any other easier way of addressing discontent. In addition, although the institutional protocol dictates that all written patient complaints received by individual clinicians should be sent to the independent complaints officer, it was not possible to check if this protocol was always followed. Therefore, the true extent of patient dissatisfaction may have been underestimated. Furthermore, those who actually decide to file a written complaint may not be a representative of the whole spectrum of patients, since they may constitute the most vindictive part of them. Spending energy to address their complaints may only solve a limited part of the entire patient dissatisfaction issue.

Third, although a response letter was sent to all patients on behalf of the head of the department, explaining and (if applicable) apologizing for the situation, there was a lack of patient feedback and information on whether the complaints were resolved.

In conclusion, written patient complaints directed to a department of radiology at a European tertiary care center are relatively few in number and have not shown a temporal increase. Knowledge of sources of patient dissatisfaction may help to reduce the number of patient complaints and improve patient care.

## Abbreviations

CI: Confidence interval
CT: Computed tomography
IRB: Institutional review board
MRI: Magnetic resonance imaging
PRISMA: Prevention Recovery Information System for Monitoring and Analysis
